# Oropharyngeal Dysphagia and Aspiration Pneumonia Following Coronavirus Disease 2019: A Case Report

**DOI:** 10.1007/s00455-020-10140-z

**Published:** 2020-06-12

**Authors:** Yoichiro Aoyagi, Miho Ohashi, Reisuke Funahashi, Yohei Otaka, Eiichi Saitoh

**Affiliations:** 1 Department of Rehabilitation Medicine I, School of Medicine, Fujita Health University, 1-98 Dengakugakubo, Kutsukake, Toyoake, Aichi 470-1192 Japan; 2 Department of Rehabilitation, Fujita Health University Bantane Hospital, Nagoya, Japan

**Keywords:** Coronavirus disease 2019, Severe acute respiratory syndrome coronavirus 2, Vagus nerve, Deglutition, Deglutition disorder

## Abstract

Cranial nerve involvement is a finding often observed in patients infected with severe acute respiratory syndrome coronavirus 2 during the pandemic outbreak of coronavirus disease 2019 (COVID-19). To our knowledge, this is the first report of oropharyngeal dysphagia associated with COVID-19. A 70-year-old male developed dysphagia and consequent aspiration pneumonia during recovery from severe COVID-19. He had altered sense of taste and absent gag reflex. Videoendoscopy, videofluorography, and high-resolution manometry revealed impaired pharyngolaryngeal sensation, silent aspiration, and mesopharyngeal contractile dysfunction. These findings suggested that glossopharyngeal and vagal neuropathy might have elicited dysphagia following COVID-19. The current case emphasizes the importance of presuming neurologic involvement and concurrent dysphagia, and that subsequent aspiration pneumonia might be overlooked in severe respiratory infection during COVID-19.

Dear Editor,

In the recent pandemic outbreak of severe acute respiratory syndrome coronavirus 2 (SARS-CoV-2) infection, referred to as coronavirus disease 2019 (COVID-19), majority of infected individuals (81%) confer mild disease, with fever, cough, and dyspnea as the most commonly reported symptoms [1]. However, for a significant minority of patients, particularly those aged over 65 years, SARS-CoV-2 infection may have very serious consequences. Relatively high proportion (20.3%) of patients requiring hospitalization requires management in an intensive care unit (ICU) [1]. Neurologic symptoms are found in 36% of patients with COVID-19 and are more common in those with severe respiratory infection (46%) [2]. However, to our knowledge, development of dysphagia has not been reported in patients with COVID-19. We herein report the first case of a patient with oropharyngeal dysphagia associated with COVID-19 and discuss the potential underlying cause.

A 70-year-old male with a history of prostate cancer and hypertension noticed that the distilled spirit shochu that he consumed daily was almost tasteless and scentless, 1 week after practicing yoga with a person who developed COVID-19 soon after. Two days later, he developed sore throat, cough, and high fever. Five days after the first symptom, he developed watery diarrhea and dyspnea. Nine days after the first symptom, the symptoms worsened and he was admitted to a university hospital. Polymerase chain reaction of the pharyngeal exudate, which was performed due to the high suspicion of COVID-19 based on the symptoms and close contact with an infected patient, was positive for SARS-CoV-2. The first computed tomography (CT) imaging revealed multiple scattered ground-glass opacities (GGO) throughout bilateral lung fields and fibrotic foci with bronchial dilatation in bilateral dorsal parts of inferior lobes. Laboratory blood tests showed a normal blood cell count with lymphopenia (3600/μL; neutrophils, 80%; lymphocytes, 16%) and an elevated C-reactive protein (19.8 mg/dL). Antibiotic treatment with azithromycin hydrate (2 g orally) and ceftriaxone sodium hydrate (2 g twice daily, intravenously) was initiated for possible superimposed bacterial pneumonia.

On the second day after admission, oxygen administration by nasal cannula was initiated. The second CT imaging on the eighth day after admission showed more dilated multiple GGO in both lungs and more advanced bilateral pulmonary foci with consolidation (Fig. 1). The patient was admitted to the ICU due to the aggravation of oxygen desaturation and dyspnea. Starting on the ninth day after admission, he was treated with a mechanical ventilator for 11 days. From the 11th day after admission, he was treated with favipiravir (1600 mg twice daily) for 13 days. On the 20th day after admission, he complained of dysphagia that he described as something stuck in his throat, and of persistent taste impairment during a meal provided after an interval of 11 days. He developed high fever of up to 39.1 °C, general malaise, cough with expectoration, and aggravated dyspnea. The laboratory tests and chest X-ray on the 21th day after admission showed elevated blood cell count (10,200/μL; neutrophils, 90%; lymphocytes, 5%) and C-reactive protein (15.8 mg/dL) as well as further enhancement of the shadows in bilateral lower lung fields. Therefore, once daily treatment with intravenous ampicillin sodium (2 g) and sulbactam sodium (1 g) was initiated for underlying superimposed aspiration pneumonia. Parenteral nutrition was continued because he could consume a limited volume of soft meals comprising rice gruel. Physical therapy using a telerehabilitation system [3] was started after his respiratory status became stable on the 36th day after admission. Testing for SARS-CoV-2 by polymerase chain reaction was negative twice; therefore, he was transferred from a depressurized room to a general ward on the 43th day and assessed for dysphagia.

**Fig. 1:**
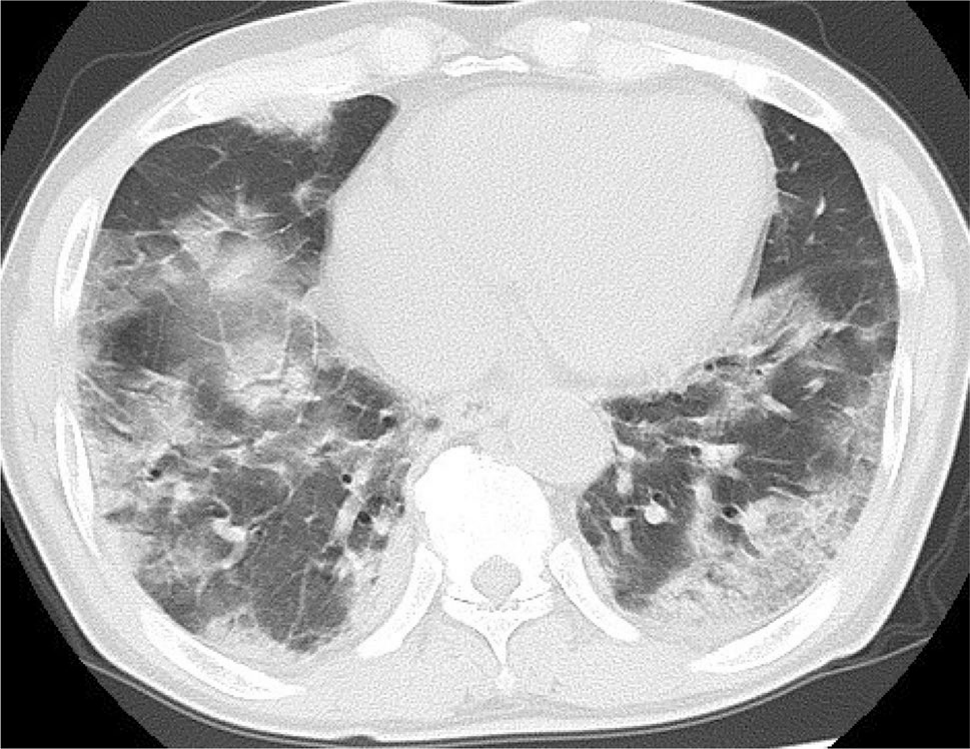
Chest computed tomography showing multiple scattered ground-glass opacities with consolidation throughout bilateral lung fields

Gag reflex was absent bilaterally. Saliva retention in the piriform recess and epiglottic vallecula was observed by videoendoscopy performed on the 45th day after admission. Touching the epiglottis or laryngeal vestibule by the fiberscope tip did not elicit a tactile sense, laryngeal reflex, or swallowing reflex. Videofluorography demonstrated that he aspirated 5 mL water that was not accompanied with coughing. Pharyngeal contraction was incomplete; consequently, large bolus retention was observed in the epiglottic vallecula and piriform sinuses. The normalized residue ratio scale (NRRS) for valleculae (NRRSv) and piriform sinuses (NRRSp) [4] were 0.55 and 0.18, respectively, following the ingestion of 5 g soft rice (Fig. 2a). High-resolution impedance manometry revealed very low mesopharyngeal peak pressure and mesopharyngeal contractile integral (56.0 mmHg and 59.1 mmHg cm s, respectively) (Fig. 2b), suggesting extremely reduced pharyngeal contractile ability at the middle pharyngeal constrictor levels, and an abnormal swallow risk index (SRI) exceeding the predictive threshold for aspiration risk (SRI > 15) [5]. Head magnetic resonance imaging showed no abnormal findings. Dysphagia rehabilitation program including tongue-hold swallow, tongue base exercise, Shaker exercise, and transcutaneous electrical sensory stimulation using interferential current was provided until the discharge home on the 65th day. The NRRSv and NRRSp slightly decreased to 0.39 and 0.09, respectively, at follow-up evaluation on the 61th day. The mesopharyngeal peak pressure, mesopharyngeal contractile integral, and SRI improved to 80.8 mmHg, 109.0 mmHg cm s, and 8.9, respectively.

**Fig. 2:**
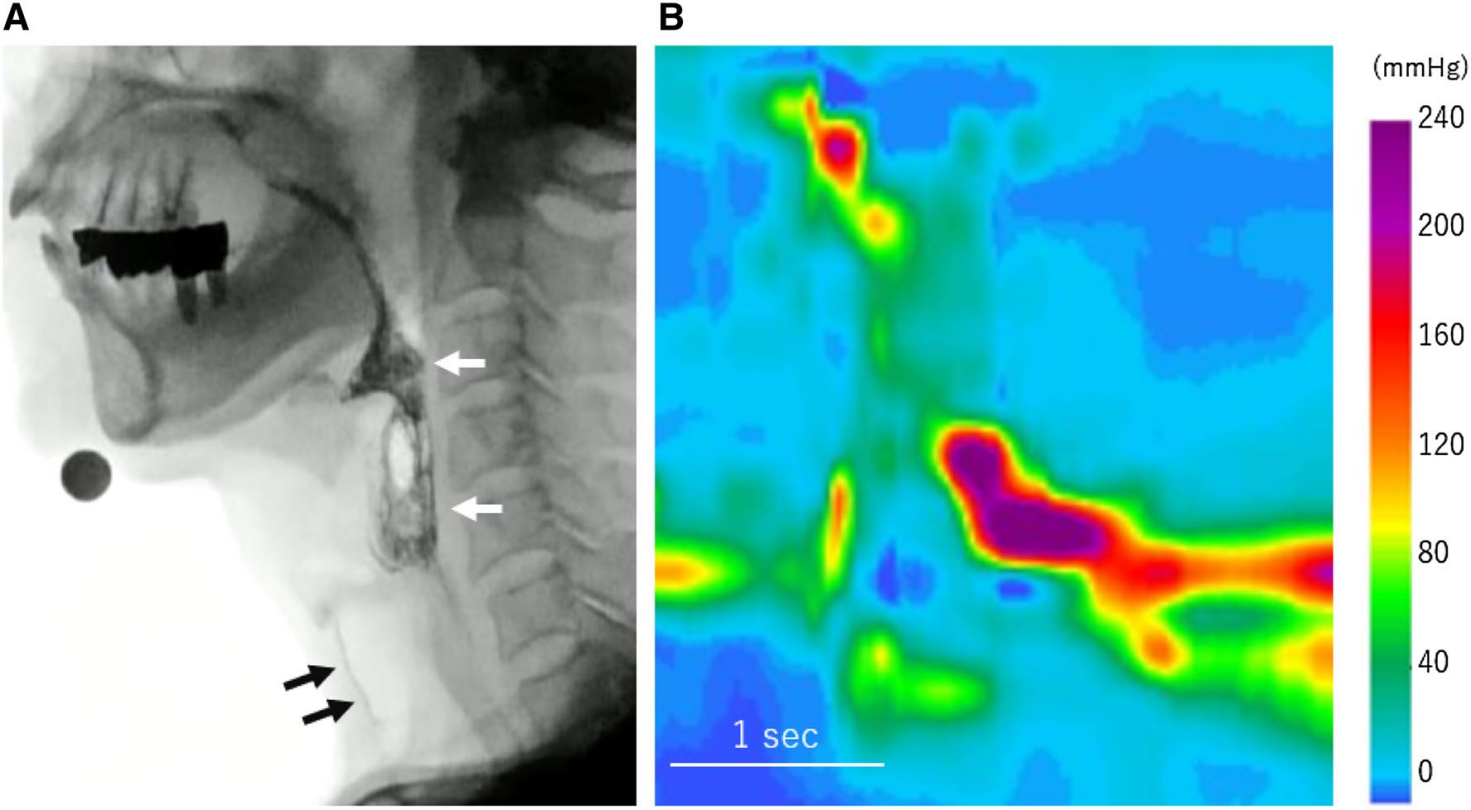
Videofluorographic image and pressure tomography obtained by high-resolution manometry. **a** Soft rice retention is observed in the epiglottic vallecula and piriform recess (white arrows), and aspirated fluid during the preceding trial is seen in the trachea (black arrows). **b** Low mesopharyngeal pressure is identified

The current patient developed oropharyngeal dysphagia and the consequent aspiration pneumonia during recovery from COVID-19. Given the concurrent presence of taste impairment, absent gag reflex, impaired pharyngolaryngeal sensation, and mesopharyngeal contractile dysfunction, glossopharyngeal and vagal neuropathy might have elicited the dysphagia in the present case. Interestingly, Mao et al*.* reported that taste and smell impairments as cranial nervous manifestations of COVID-19 were observed in 5.6% and 5.1% of hospitalized patients, respectively, and that the rate of skeletal muscle injury was 10.7% [2]. Skeletal muscle injury or atrophy was less likely in the present case due to the absence of muscle pain, elevated serum creatine kinase, and sarcopenia-related symptoms. Alternatively, dysphagia associated with prolonged endotracheal intubation might have aggravated the swallowing difficulty [6]. This first reported case of unusual dysphagia likely due to the involvement of glossopharyngeal and vagal nerves following COVID-19 emphasizes the importance of presuming neurologic involvement and concurrent dysphagia and that subsequent aspiration pneumonia might be overlooked in severe respiratory infection during COVID-19.
